# A Novel Thin NIPAM Gel Cassette Dosimeter for Photon-Beam Radiotherapy

**DOI:** 10.1371/journal.pone.0031836

**Published:** 2012-03-12

**Authors:** Ling-Ling Hsieh, Kai-Yuan Cheng, Bor-Tsung Hsieh

**Affiliations:** Department of Medical Imaging and Radiological Sciences, Central Taiwan University of Science and Technology, Beitun District, Taichung City, Taiwan, Republic of China; University Health Network, Canada

## Abstract

The response of thin polymer gel cassettes (called NIPAM gels) to ionizing radiation was investigated in this study. The NIPAM gels were prepared from gelatin, N-isopropyl acrylamide, tetrakis (hydroxymethyl) phosphoniumchloride, and N,N′-methylene-bis-acrylamide. Gel cassettes were irradiated in a phantom using a linear accelerator, and the polymerization morphology of irradiated NIPAM gel was characterized using scanning electron microscopy. The dose-response sensitivity of the NIPAM gels was evaluated using the differences in optical densities. The optical densities were obtained using a computer-controlled CCD camera that was connected to a planar illumination source for acquisition of optical transmission images. The central axis depth dose profiles of the phantom were extracted, and a comparison with ionization chamber measurements demonstrated similarities in profiles. The sensitivity, linearity of the response, accuracy, and reproducibility of the polymer gel cassettes were acceptable. However, the profiles of the half-blocked field irradiation showed no significant dispersion in the visible region. This study also extensively investigated the spatial stability of the NIPAM gel. The results showed that the gel cassette response remains stable for up to three months after irradiation.

## Introduction

Recent advances in radiotherapy have placed great importance on the development of dosimetric techniques [1]–[3]. Treatments such as intensity-modulated radiotherapy and stereotactic radiosurgery optimize the dose to the tumor volume while minimizing the dose to normal healthy tissues and surrounding critical structures, such as the optic chiasm, brain stem, and spinal cord. These treatments must be performed correctly and accurately because they are potentially dangerous and painful and may result in serious complications. Therefore, complete verification and 3D dosimetric techniques are required before treatment [4], [5]. A significant development occurred in 1993 [6] with the proposal of a polymer gel dosimeter. Ionizing radiation induces a number of free radicals from water hydrolysis to initiate a polymerization reaction of the monomers. The resulting polymer particles do not diffuse through the gel matrix, and the diffusion limitation that is associated with Fricke gels is overcome [7]. The two major advantages of polymer gel dosimeters are their ability to determine the integrated 3D dose distribution and their ability to adopt different shapes [8]. Polymer gel dosimeters are in the first phase of experimentation and require further improvement. In fact, good consistency of images after irradiation has been obtained (up to months), but the poor stability of the unirradiated gel matrix results in a strong dependence of the dosimeter response on the time between preparation and irradiation. Senden et al. proposed a new polymer gel, based on N-isopropyl acrylamide, which is a less toxic monomer and which he named the NIPAM polymer gel [9]. Since then, several studies on the development and improvement of polymer gels have been conducted. The dose response of the NIPAM gels is linear over a wide dose range. The polymerization process is also highly correlated to oxygen inhibition; the antioxidant tetrakis (hydroxymethyl) phosphonium chloride (THPC) reacts with the gelatin but not with the crosslinker N,N′-methylene-bis-acrylamide (Bis) [10]. The characteristics of the NIPAM gel (i.e. sensitivity, linearity, and temporal stability attainment) at various dose ranges necessitate further investigation for clinical treatment [11].

An ideal gel dosimeter and readout system should have a highly sensitive dose response with good linearity as well as low energy and dose rate dependence. Researchers have investigated several readout modalities in an attempt to obtain dose information from the polymer gel. Recently, approaches included magnetic resonance imaging (MRI) [12], optical computed tomography (OCT) [13], [14], x-ray computed tomography (CT) [15], and ultrasound [16]. It has been shown that MRI as a readout system has great potential to be a gel dosimeter in clinical radiation therapy. The weaknesses of MRI are its long image acquisition time and temperature sensitivity of the transverse relaxation time (T_2_). Its introduction into the clinical routine has been attempted but has often failed due to the high cost and low availability of imaging time on MRI scanners. Alternative readout methods, such as OCT systems with spatial resolutions of up to 100 µm [17], provide an inexpensive and easy-to-use alternative imaging modality. Their spatial resolution is based on the optical attenuation coefficient (or optical density per unit length), which depends on the degree to which the polymer micro-particles scatter light, and it is proportional to the absorbed dose. However, OCT approaches introduce novel challenges because of the inherent complications from index of refraction matching at interfaces, multiple scattering in polymer systems, etc. [18]–[20]. A convenient readout method is characterized by a combination of a plane illuminator and a non-uniform pixel sensitivity of the low-cost CCD camera. The method uses a CCD camera to detect grey-level (GL) images of the light, which are transmitted by gel dosimeters before and after irradiation. Although the optical analysis system has a spatial resolution of only 38 pixel/cm, which is less than OCT [21], it can be performed with a simpler and less expensive instrument. The suitable optical imaging apparatus is transportable and can be placed near the irradiation unit. Therefore, it is possible to complete a dosimeter analysis, as detection of all images only takes a few minutes.

The present study evaluated the feasibility of a thin-cassette design on a normoxic polymer gel dosimeter using NIPAM monomers. Gel dosimeters in the form of thin cassettes create the possibility of obtaining spatial dose distributions and measurements of each dose contribution in a radiation field. These advantages arise from the cassette's geometry, which results from stacking to obtain a solid phantom. This is convenient because thin gel cassette transmission images are detectable using a simple and reliable optical analysis.

## Materials and Methods

### Thin gel cassette preparation and irradiation

#### 1. Thin gel cassette preparation

**Figure 1:**
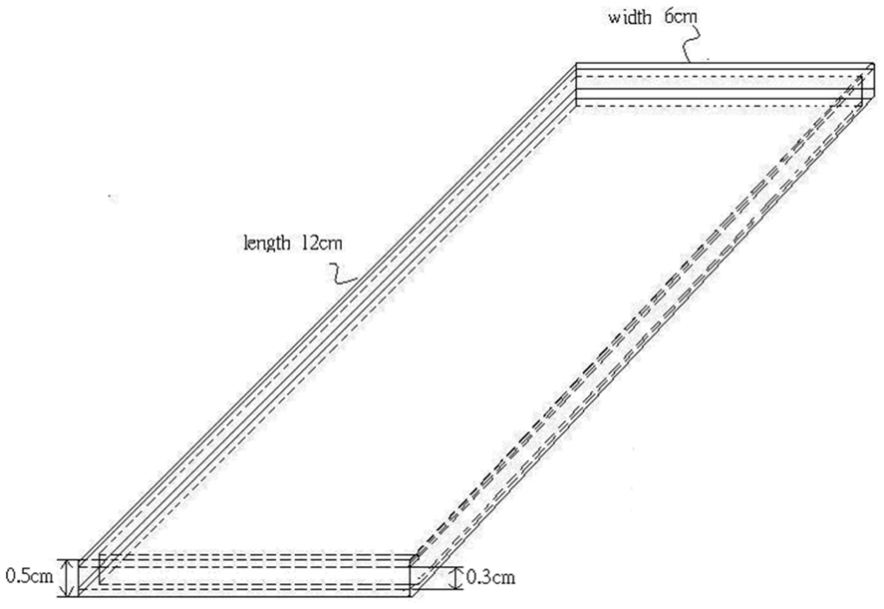
Thin cassette dosimeter containers. The cassette dosimeter consisted of two 1 mm thick transparent polymethylmethacrylate sheets with a 12×6 cm^2^ surface area.

The adopted NIPAM gel dosimeters were in the form of thin cassettes (Fig. 1) with a 3 mm thickness and a 12×6 cm^2^ surface area. The gel was contained in structures that consisted of two transparent polymethylmethacrylate sheets with a 1 mm thickness each. NIPAM gels were prepared by using 5% (all percentages by weight) gelatin (porcine, 300 Bloom Type A, Sigma-Aldrich) and allowing it to swell at room temperature in 87 wt% de-ionized water for 5 min (22±1°C). It was then heated to 45°C. As a result, the gelatin solution became clear and transparent. While stirring continuously, 3% N,N′-methylene-bis-acrylamide (Merck) and 5% NIPAM(97%, Sigma-Aldrich) were combined and heated at 45°C, taking approximately 15 min to dissolve. Subsequently, 10 mM THPC (Sigma-Aldrich) was added to the solution as an antioxidant, and the solution was mixed for 2 min. Using a syringe, the NIPAM gel solution was introduced into holding structures of cassette through a small hole on top of gel cassette, before it started to gel. The gel cassette is composed of a 3 mm frame between two transparent sheets. The hole was sealed with silicone, to prevent leakage of oxygen into the gel. Finally, the thin gel cassette was wrapped with aluminum foil and stored in a refrigerator at 4°C to prevent light-induced pre-polymerization.

#### 2. Irradiation

Gel cassettes were irradiated under graded doses for approximately 6 hours after they were manufactured, using the 6 MV photon beam from the Clinac 18 Varians linear accelerator (Linac) at a dose rate of 400 MU sec^−1^. The gel cassettes were inserted into a phantom, which is a cube composed of polystyrene layers with a space that is appropriate for gel cassette dosimeter insertion. Irradiations were performed under 10×10 cm^2^ and 1×1 cm^2^ fields at a source-surface distance of 100 cm. After irradiation, the gel cassettes were stored in a refrigerator for subsequent measurements.

Depth dose profiles in a cubic polystyrene phantom (20 cm side) were detected at the center of a 10×10 cm^2^ and 90° gantry angle irradiation, whereas the source-surface distance was 100 cm, the prescribed dose at the isocenter was 10 Gy. In this study, the dose-optical density difference Δ(OD) calibration curve was obtained by fitting the central axis depth–optical density difference Δ(OD) curve to the depth–dose profiles. The depth-dose profiles were measured in a separate experiment by scanning a 0.6 cm^3^ ionization chamber (PTW-30013, Farmer type, Germany) under the same conditions.

### Oxygen contamination

The effects of oxygen were studied in three gel cassettes that were filled and stored in a refrigerator at 4°C. These samples were not sealed at the upper border of the thin gel cassette, to expose the gels to air before irradiation. The gel cassettes were irradiated with up to 10 Gy, and all gel cassettes were imaged simultaneously 24 h after irradiation in a clinical 1.5 T MRI scanner (Siemens Symphony Tim system, Germany) with a multi-echo spin echo protocol. *R*_2_ ( = 1/T_2_) maps were calculated using MATLAB® software.

### Instrumentations for thin gel cassette measurements and data analysis

Measurements were performed after irradiation. The gels were stored in a refrigerator at 4°C for 12 hours. The analytical instruments consisted of a planar illumination source, a CCD camera (12 bit, 4096 shades of gray) for the acquisition of optical transmission images, and a computer for CCD control and the storage and processing of acquired images. Gel cassettes were placed approximately 35 cm away from the lens and an interference filter was placed between the 48 mm camera lens and the CCD detector, to match the wavelength (∼630 nm) of the absorption peak. The images were analyzed within a 6×12 cm^2^ area, which included the gel cassette.

In CCD images, the matrix size covering the entire gel cassette is approximately 620×310. Therefore, the CCD system used in this study has a spatial resolution of ∼205 µm, whereas the maximum spatial resolution is typically 154 µm.

The following equation [22] reveals the absorbed dose, which was deduced from the light transmittance at ∼630 nm before and after irradiation:
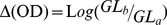
(1)Where Δ(OD) is the difference in the optical density and GL_a_ and GL_b_ are the gray-level (GL) values of the images detected before and after irradiation, respectively. Specific software was developed in the programming environment MATLAB (The Math Works Inc., Natick MA, USA) to identify the dose profiles.

### Morphological analysis

The morphological details of freeze-dried specimens were examined using scanning electron microscopy (SEM) (JEOL T330A) with an acceleration voltage of 15 kV. Specimens were coated with a gold metal layer to provide proper surface conduction.

## Results and Discussion

### Photography of radiation exposure

**Figure 2:**
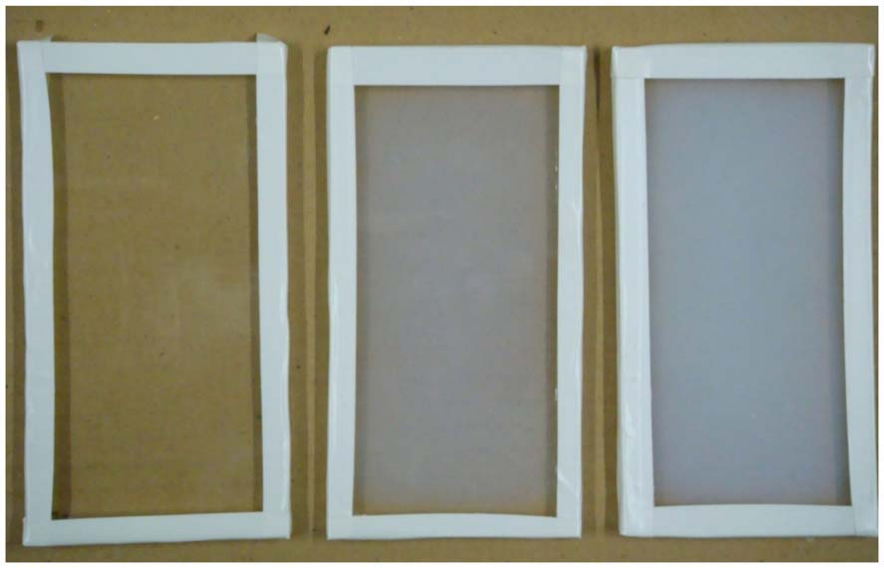
Thin polymer gel cassettes after irradiation. From left to right: 0 Gy (before irradiation), 5 Gy, and 10 Gy.

The NIPAM gel cassettes at two different absorbed radiation doses are shown in Fig. 2. The different radiation dose during the polymerization was an obvious factor for the quantity of crosslinked polymers. Based on the dose dependence of the NIPAM gels, the intensity of radiation at 10 Gy had a greater effect than radiation at 5 Gy.

### Morphological analysis

**Figure 3:**
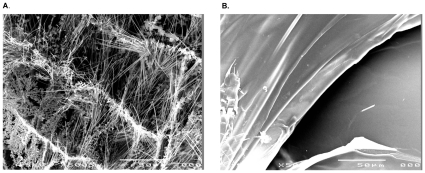
SEM micrographs of the NIPAM gel. A. non-irradiated NIPAM gel. (lateral view). B. irradiated NIPAM gel under a 10 Gy radiation dose. (lateral view).

SEM determines the surface morphology of polymer gels with radiation polymerization. Microphotographs of the cross-sectional NIPAM gel samples at 500× are shown in Fig. 3. Non-irradiated NIPAM gels revealed a fibrous morphology (Fig. 3A). This fibrous structure gradually disappeared, and the layer structure reappeared after irradiation (Fig. 3B).

### Dose response and depth dose distribution

**Figure 4:**
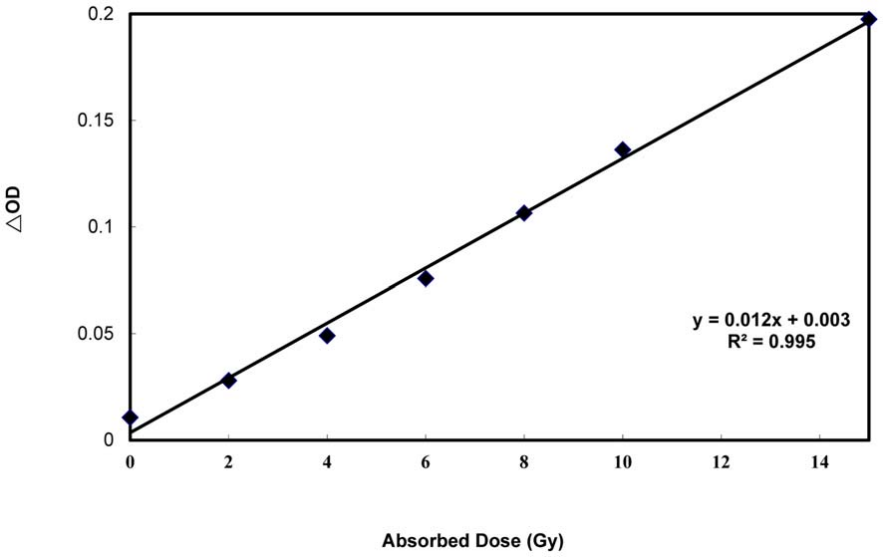
Variation in the optical density difference Δ(OD) versus dose. The slope represents a sensitivity of 0.012 Gy^−1^ from 0 Gy to15 Gy.

Fig. 4 demonstrates the good linearity of the dose vs. the optical density differences Δ(OD) from 0 to 15 Gy. The slope represents a sensitivity of 0.012 Gy^−1^.

**Figure 5:**
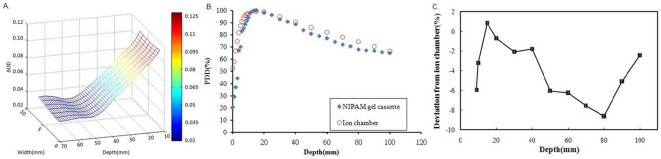
Comparison between the depth-dose profiles of the NIPAM gel cassette and the ionization chamber. A. Depth-dose profiles extracted from the Δ(OD) center on the surface of the 3 mm-thick NIPAM polymer gel cassette. B. On-axis percent depth dose profiles of the NIPAM gel cassette and the ionization chamber in the phantom after exposure to radiation of 10 Gy. C. Dose differences between gel and ion chamber measurements for the curves in B.

The percent depth dose (PDD) profile of the NIPAM gel cassettes was obtained, and compared between the ionization chamber dosimeters (Fig. 5). The images of Δ(OD) that correspond to 3 mm thick polymer gel cassettes are shown in Fig. 5A. The on-axis PDD profiles of the NIPAM gels are compared with the profiles from the ionization chamber dosimeter (Fig. 5B). The experimental dose profiles in the polymer gel cassettes were similar to the ionization chamber depth dose profiles, which showed a general effect of polymerization. The calculated maximum percentage depth dose of NIPAM gel is close to the measured PDD curves of calibrated ionization chambers. We evaluated the accuracy of the depth dose profiles with gel cassettes and an ionization chamber. The results for the gel cassettes are shown in Fig. 5C and have an average absolute dose discrepancy as low as 4.07%. The energy and dose-rate dependence of the NIPAM gel, assumed in the literature [23],The maximum sensitivity difference was reported to be 33% for dose rates from 100 to 500 cGymin^−1^ and 10% for X-ray energy from 6 MV to 15 MV. Although the energy dependence is minor, there is a slight dose rate dependence. However, good correspondence is obtained with the CCD camera readout, as evidenced by the small discrepancy with respect to the reference depth dose profiles.

**Figure 6:**
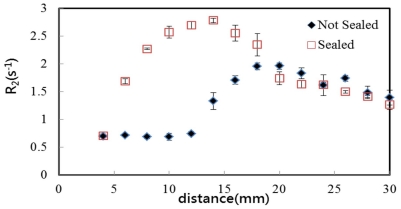
R_2_ profile of gel cassette dosimeters unsealed at the upper border, which were exposed to air before irradiation. Compare oxygen containment in gel cassettes dosimeters sealed and unsealed at the upper border and exposed to air. Cassettes were stored in a refrigerator at 4°C before irradiation and were scanned 24 h after irradiation. R_2_ profiles are derived along the cassette axis, each gel cassette sample received a dose of 10 Gy.

Fig. 6 illustrates spin-spin relaxation rate (R_2_) values derived from gel cassettes, which had not been sealed at the upper border to allow for exposure to air before irradiation. Oxygen contamination inhibited front polymerization of the NIPAM gel cassette, resulting in a border effect that was almost 14 mm in width. This occurs as a uniform baseline with an R_2_ of approximately 0.7 s^−1^. Beyond this point, the concentration of oxygen declines to values below the amount required for complete inhibition, and R_2_ rapidly increases to a maximum 2.0, which results in lower maximum R_2_ values than those in cassettes sealed at the upper border. The oxygen contamination can have a significant influence on the R_2_ response. The percentage differences between maximum R_2_ values for cassettes not sealed or sealed at the upper border is approximately 28.6%, which may translate to a maximum dose discrepancy of up to approximately 60% depth dose at low depths, when using ionization chamber calibration. The results in this study were similar to those previously reported in the literature [24]. Therefore, a border effect was detected because the dosimeters were not well-sealed. Some oxygen entered into the gel cassette and inhibited the polymerization, which resulted in the consequent local loss in sensitivity [25].

### Temporal stability of the NIPAM gel cassettes

**Figure 7:**
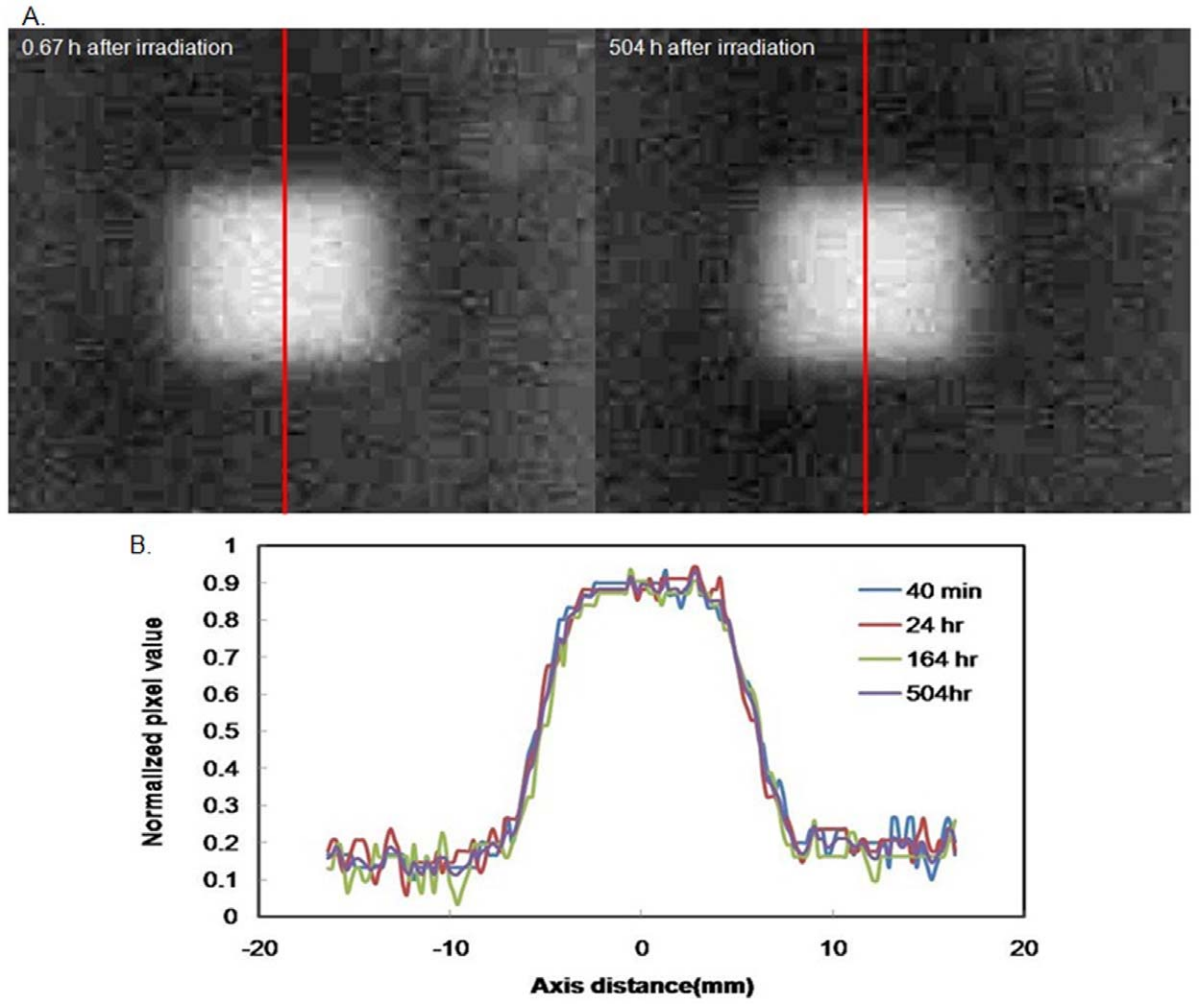
CCD camera image and profiles for the same gel cassette exposed to 15 Gy at different time post-irradiation. A. CCD camera image relative to 0.67 h and 504 h after irradiation. B. The dose intensity in the central region of an irradiated gel cassette was observed for doses equal to 15 Gy and measured 0.67, 24 h, 164 h, and 504 h after irradiation.

The temporal stability of the gel cassettes after irradiation is indicative of their clinical applicability. Differences in sensitivity and linearity were measured at various periods after irradiation. Table 1 shows the temporal variation of the gel cassettes, which had a deviation in the sensitivity of less than 5% at almost 90 days post irradiation. In addition, the deviation in the linearity of the gel cassettes was less than 1% at almost 90 days post-irradiation. According to Senden et al. [9], the chemical reaction may persist for up to 24 h after irradiation because of the continuous polymerization reaction that is caused by the long-lived radicals. The results of the current experiment demonstrated that the stability of the NIPAM gel persisted for up to three months post-irradiation. Furthermore, Fig. 7 presents the CCD camera images and profiles for the same gel cassette exposed to 15 Gy at different times post-irradiation. The intensity around regions of the field was 0.89 (normalized pixel value). The values of the original response (created by exposure to the radiation) dropped slightly to an average value of 0.86 (normalized pixel value) after 504 hr. Contrary to the enhancement at the edge, a decrease of only approximately 3.3% was observed in the apparent response at the center of the radiation field. Therefore, the NIPAM gel appears to be very stable.

**Table 1:** Temporal stability of the NIPAM polymer gel.

Days after irradiation	Linearity(R^2^)	Difference in linearity (%)	Sensitivity	Difference in sensitivity (%)
1	0.9951	0%	0.0129	0%
2	0.9964	0.13%	0.0125	3.70%
3	0.9924	0.27%	0.0133	3.10%
9	0.9972	0.21%	0.0128	0.77%
15	0.9926	0.25%	0.0134	3.88%
30	0.9954	0.03%	0.0130	0.76%
60	0.9878	0.73%	0.0123	4.65%
90	0.9860	0.91%	0.0125	3.10%

### Spatial stability

**Figure 8:**
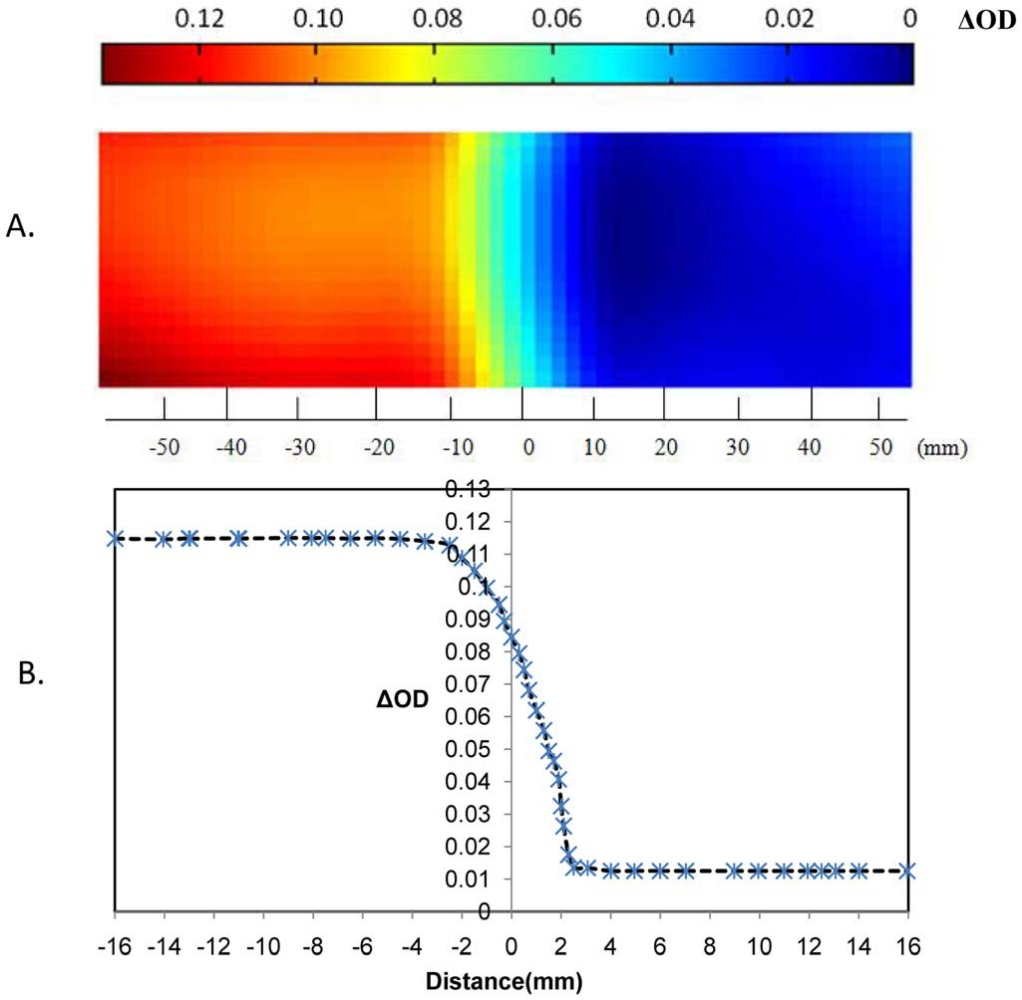
Image of a partially irradiated thin polymer gel cassette. A. Image of a partially irradiated polymer gel cassette. B. Dose profiles in the central axis.

The dose profiles of the samples were half-block field-irradiated to assess the spatial stability of the NIPAM gel cassettes (Fig. 8). Profiles of the NIPAM gel dosimeter across an irradiated/non-irradiated transition border (longitudinal direction) of a half-blocked field were assessed. The profiles exhibited an edge aggregation of approximately 5 mm when irradiated to 10 Gy. Polymerization pushes the edge of the boundary of the irradiated region toward the non-irradiated region, which leads to a narrowing of the penumbra [26]. No significant dispersion in the visible region was detected.

### Conclusions

The utilization of NIPAM gel cassettes as photon-beam dosimeters demonstrated good linearity from 0 Gy to approximately 15 Gy. Moreover, the optical response of the NIPAM gels remained unaltered for at least 90 days after irradiation, and no significant enhancement of the edge of radiation field was observed. The dose profiles of the gel cassettes reveal a good correspondence when obtained with CCD camera readout. This is evident from the small discrepancy with respect to the reference depth dose profiles from the ionization chamber, thereby confirming the reliability of this method.
